# Supplementary material for: Evaluation of Antimicrobial Activity in the Extract of Defatted Hermetia illucens Fed Organic Waste Feed Containing Fermented Effective Microorganisms
Source: Animals (Basel). 2022 Mar 8;12(6):680. doi: 10.3390/ani12060680 (PMC8944688; doi:10.3390/ani12060680)
Supplement: Supplementary file 1 [file animals-12-00680-s001.zip › animals-1592119-supplementary.pdf]

Supplementary information

Table S1. Comparison of nutritional composition in HIL and crushed HIL powder.

Composition	Moisture (%)	Crude carbohydrate (%)	Crude protein (%)	Crude fat (%)	Crude ash (%)
HIL	0.57 ± 0.15	2.84 ± 0.87	8.63 ± 0.98	86.66 ± 1.57	1.30 ± 1.62
Crushed HIL powder	1.19 ± 0.24	20.90 ± 4.66	64.00 ± 4.62	7.17 ± 0.73	10.67 ± 4.60
